# Ontology Based Document Enrichment in Bioinformatics

**DOI:** 10.1002/cfg.141

**Published:** 2002-02

**Authors:** Robert Stevens

**Affiliations:** Department of Computer Science University of Manchester Oxford Road Manchester M13 9PL UK

## Abstract

Controlled vocabularies are common within bioinformatics resources. They can be used to give a summary of the knowledge held about a particular entity. They are also used to constrain values given for particular attributes of an entity. This helps create a shared understanding of a domain and aids increased precision and recall during querying of resources. Ontologies can also provide such facilities, but can also enhance their utility. Controlled vocabularies are often simply lists of words, but may be viewed as a kind of ontology. Ideally ontologies are structurally enriched with relationships between terms within the vocabulary. Use of such rich forms of vocabularies in database annotation could enhance those resources usability by both humans and computers. The representation of the knowledge content of biological resources in a computationally accessible form opens the prospect of greater support for a biologist investigating new data.


**Comparative and Functional Genomics**
*Comp Funct Genom* 2002; **3**: 42–46.
**DOI:** 10.1002/cfg.141

**Conference Review**

### Ontology based document enrichment in bioinformatics

Robert Stevens*
*Department of Computer Science, University of Manchester, Oxford Road, Manchester, M13 9PL, UK*

*Correspondence to: Department of Computer Science, University of Manchester, Oxford Road, Manchester, M13 9PL, UK. E-mail: robert.stevens@cs.man.ac.uk*

Received: 6 December 2001
Accepted: 6 December 2001
Published online: 15 January 2002

### Abstract

**Controlled vocabularies are common within bioinformatics resources. They can be used to give a summary of the knowledge held about a particular entity. They are also used to constrain values given for particular attributes of an entity. This helps create a shared understanding of a domain and aids increased precision and recall during querying of resources. Ontologies can also provide such facilities, but can also enhance their utility. Controlled vocabularies are often simply lists of words, but may be viewed as a kind of ontology. Ideally ontologies are structurally enriched with relationships between terms within the vocabulary. Use of such rich forms of vocabularies in database annotation could enhance those resources usability by both humans and computers. The representation of the knowledge content of biological resources in a computationally accessible form opens the prospect of greater support for a biologist investigating new data. Copyright © 2002 John Wiley & Sons, Ltd.**

**Keywords:** Ontology; controlled vocabulary; annotation; document enrichment

### Introduction

Within the bioinformatics arena, there has been much interest in ontologies over the past few years [7]. It is almost true that this interest has been matched by confusion as to the meaning of ontology; what counts as an ontology; and to what uses an ontology can be put. In this article I will discuss these points with respect to the use of controlled vocabularies within bioinformatics resources and the relationship of such vocabularies to ontology.

Ultimately, my message will be less about an exact definition of an ontology, but more about how good the ontology is in terms of its fitness for purpose. First, I shall discuss the meaning of ontology (see 'What is ontology?'), particularly in the context of a computer science understanding of the term. The second topic in this section is a brief discussion of what artifacts could be included under the concept of ontology. The main argument of the paper will be about the uses of controlled vocabularies; their uses and how ontologies provide a means for increasing their utility and enriching our documents by making their knowledge computationally accessible (see 'Document enrichment'). This article will conclude with a brief discussion of the points raised.

### What is ontology?

Ontology is an old philosophical term borrowed by modern computer science and in doing so, the meaning has somewhat changed. Originally ontology is the study or concern about what kinds of things exist – what entities or 'things' there are in the universe [3]. In broad terms this is a description of that which we know. The modern definition of ontology used in computer science is 'a shared understanding of a domain that is processable by both computers and human beings'. In essence, the only change is that we wish our ontologies to be processable by computers; we might also be slightly narrower, in that we talk about a domain, not the Universe. The core definition of ontology is the capturing of knowledge of a domain; everything else is adornment, that practitioners use to discriminate between ontologies – especially their representation style.

Knowledge is that which we understand about an

Copyright © 2002 John Wiley & Sons, Ltd.

'thing' or entity in a domain. We may, for instance, wish to capture knowledge about the entity **Enzyme**; we might say that it is a kind of **Protein**, with the property of catalysing a **Reaction**. the reaction itself has **Substrates** and **Products** and may involve a **Cofactor** or two. This (and more) is what we understand about the concept of **Enzyme** in the domain of molecular biology.

Uschold [9] has a useful definition of the computer science definition of an ontology:

> An ontology may take a variety of forms, but necessarily it will include a *vocabulary of terms*, and some *specification of their meaning.* This includes definitions and an indication of how concepts are inter-related which collectively impose a structure on the domain and constrain the possible interpretations of terms.

What people choose to call 'ontology' runs a spectrum from a list of word; through glossaries and thesaurae, database schema; simple taxonomies; to artifacts built using knowledge representation languages such as Frames and Description Logics [7]. All these forms capture knowledge; at least to some extent. All create some sort of shared understanding of a domain of interest. What is more in doubt is the provision of specification of those terms and the embedding of terms within a structure of relationships. These relationships from one concept to another are one way in which the properties of a concept can be described. These relationships give ontologies some of their utility.

The line dividing ontology from non-ontology is really in the eye of the beholder. I think an ontology needs three components:

(i) A collection of terms representing concepts in the domain;
(ii) Definitions of the meanings of those concepts, either in the form of properties or natural language descriptions; and
(iii) the concepts held within a lattice of relationships, including taxonomic, is a kind of, partitive, ..., relationships, that place one concept within a context of other concepts.

A controlled vocabulary is often understood simply to be a collection (a set or list) of terms. There is some regulating authority that states which terms can be used. the working definition of ontology used here adds definition of terms and a structure of relationships that support interpretation to this simple idea of controlled vocabulary. An ontology can, however, be used to deliver a controlled vocabulary. Using an ontology to give a controlled vocabulary for annotation also delivers definitions of terms used and, as we will see later, a structure that can be exploited for processing the knowledge in a resource.

### Use of controlled vocabularies

Controlled vocabularies are used by annotators to indicate values for certain attributes of the content of a resource. Bates [2] talks of bringing the annotator and investigator closer together. Bates describes how the annotator has a deeper knowledge or understanding of the annotated entity than the person querying the database and that the controlled vocabulary brings the investigator closer to that understanding. This model may not be wholly accurate in bioinformatics, where an investigator will have a range of understanding of the entity (a protein, for instance) from a profound understanding of a domain to the merely superficial. Bates'view about bringing annotator and investigator closer does, however, remain a strong point; it is the shared understanding that is important. The Gene Ontology (GO) [8] is used to annotate gene products as to their molecular function, biological process and cellular location. When the same ontology can be browsed by an investigator to retrieve gene products, they are using the same resource as the annotator and, it is hoped, the ontology creates a shared understanding of the domain that brings the two classes closer together.

Controlled vocabularies are really used for what the name implies. They are a collection of terms and use in certain situations is restricted to those terms. What this means is that a partial shared understanding is created. For instance, when the term 'receptor' is used in a SWISS-PROT [1] keyword field, that term is the only word allowed to denote that concept – other synonyms are not allowed. This shared understanding may be only *partial* as the terms may not be defined, nor placed within a context of inter-term relationships. SWISS-PROT keywords have recently been defined **http://www.expasy.ch/cgi-bin/keywlist.pl**. The definition for the keyword 'Activator' is:

Copyright © 2002 John Wiley & Sons, Ltd.

Protein that positively regulates either the transcription of one or more genes, or the translation of mRNA.

Such definitions will aid consistent use by annotators.

Other controlled vocabularies in SWISS-PROT, such as the feature table keys, comment topic keys and the terms used in the organelle and reference coverage fields are defined (see **http//ca.expasy.org/sprot/userman.html#ftregion** for feature key definitions), so the shared understanding is greater.

What these controlled vocabularies tend to lack is the richer structure demanded by many ontologists. The SWISS-PROT keywords are just a list of terms. the feature table keys do, however, have an implicit taxonomic relationships within groups of keys. For example, there are various kinds of 'region' in the SWISS-PROT feature keys. The key 'site', itself arguably a kind of region, can be seen to have a child 'active site'. The feature 'region', is also split into the child 'binding region' itself divided into 'nucleotide binding, DNA binding' and 'metal binding'. The 'zinc finger' feature is a kind of DNA binding site, but also a kind of metal binding site. This implicit taxonomy and other relationships can be seen in Figure 1. In most systems that use such keys, such as SRS [5], this implicit structure is not exploited. A query against SWISS-PROT for the feature key metal would retrieve only those features labelled as metal, but not those labelled **ca-bind** and **zn-finger**, which are also really kinds of metal binding site. In the following section I will describe how the structure offered by an ontology can help both the annotator and the user of a bioinformatics resource.

### Document enrichment

I have already argued that a controlled vocabulary, whether or not delivered by an ontology, helps create a shared understanding (or partially so) of a domain. The argument now moves on to how a an ontology rich in relationships can be exploited in bioinformatics applications. A rich ontology can be used to check annotation in a database entry. The Gene Ontology currently exists as three separate taxonomies of term; there are no relationships between the molecular function, biological process and cellular location ontologies. the annotation process relies upon the biological expertise of the annotator. Such relationships could define which functions took part in certain processes and where in a cell or outside a cell these activities took place. this extra knowledge could then be used to check or control the annotations made in compiling an entry. The application could guide the annotator through

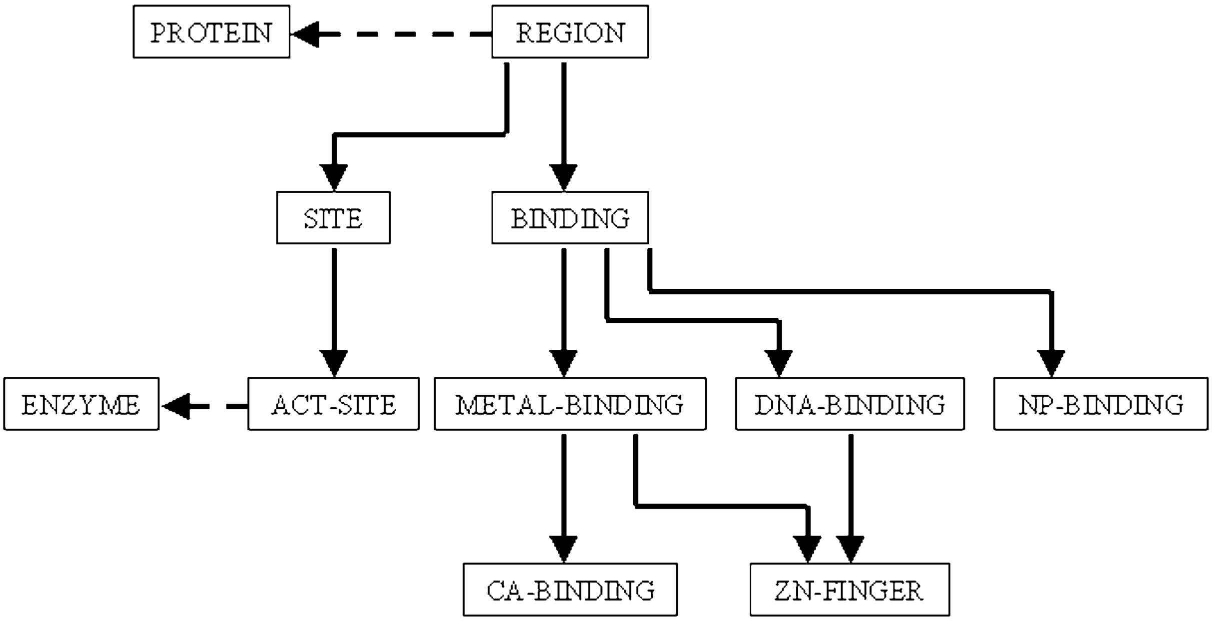

**Figure 1.** The implicit taxonomy and other relationships found in the SWISS-PROT feature keys for protein regions

Copyright © 2002 John Wiley & Sons, Ltd. 

the process of marking up an entry and prevent mistakes occurring. It is possible, though unlikely, that a human annotator could give a protein the attributes of a 'transfer RNA' function; 'amino acid biosynthesis' and 'extra cellular matrix' from within the Gene Ontology. this is clearly biological nonsense, but more subtle and harder to detect errors could creep into annotation by attributing inappropriate terms to gene products. The knowledge captured in the ontology about what functions are present in processes in which location, allows a computational check to be made upon annotations.

It would also be possible to add relationships between terms and the species in which they occurred. Our ontology, for instance, would want to say that the process of photosynthesis occurs in plants, but not in mammals. Again, this knowledge is obvious, for the sake of argument, but our ontology could talk about the biological process in which phospholipases act, including that they are part of snake venom. An expressive knowledge representation language can restrict the action of phospholipase in venom to certain species, without affecting the general application to other species. The counter argument is the complexity of biology and the difficulty in modelling that knowledge [6] – there are often exceptions to knowledge, that, for example, avian red blood cells contain nuclei, and these have to be captured in an ontology.

This richer structure can aid querying. The implicit structure seen in SWISS-PROT feature keys (see Figure 1) could be exploited during the querying of the databank. Asking for all metal binding sites would usually only retrieve those with the feature key 'metal', but exclude those with the key 'ca-bind' and 'zn-finger'. Calcium binding sites are obviously kinds of metal binding sites and should be included in the answer. This is not so much a failing of SWISS-PROT, but a failing in the applications that query the resource. this would, however, be easier if the relationships between types of feature were explicit. consultation of the ontology during query answering would ensure complete recall of all metal binding sites.

This exploitation of structure can be pursued further. Returning to the example of the Gene Ontology above, where the three taxonomies have been interlinked within relationships capturing knowledge about, for instance, the processes in which functions act, can be used to ask complex biological queries within a databank. it would be possible, for example, to ask 'find the proteins that have a calcium binding site, are involved in signal transduction and located in the cell membrane of mice'. Also, given a set of answers to a query, it would be possible to use this structure to navigate around the structure of an ontology from a feature like a binding site to functions and processes implied by that feature.

As well as more constrained annotation like features, ontology could supplement the more 'free language' style of SWISS-PROT comment fields. the ontology would not necessarily replace the textual annotation, as it is still desirable that humans can read the annotation. Nor would it be possible to completely represent the richness possible in natural language. use of ontology supplemented annotation can aid machine processing (as described above) by capturing the knowledge in a form processable by computers.

The Gene Ontology describes function, process and location without any modification as to its validity. Many annotations talk about the function ascribed to a gene product as 'experimentally validated' or 'by similarity' or 'probable'. In its present form, GO could not perform all these tasks. There is no reason, however, why it should not do so in the future. Validity and evidence could be included as part of the ontology, rather than a separate collection of terms used by annotators. A relationship would be made, for instance, between **Molecular-Function** and evidence. A dynamic or post-co-ordinated, compositional ontology [4] would allow a modifier such as **Experimentally derived** to be composed with any molecular function such as **ATP dependent calcium ion channel**, creating the concept of **Experimentally derived ATP dependent calcium ion channel**, without having to exhaustively create all possible concepts implied by the relationship. So, a rich series of annotations can be made, linking a protein to its molecular function and thence to the process involved, its cellular location, species and the degree of validity the annotation holds. Such annotations can hold almost as much information as the textual fields of databases; making an equivalent amount of knowledge computationally accessible.

### Summary

The use of ontologies has become increasingly popular in bioinformatics – for annotation, querying, database schema and community reference [7].

Copyright © 2002 John Wiley & Sons, Ltd. 

The important features of an ontology are that they capture domain knowledge in a computationally accessible form. A full and rich ontology has a vocabulary of terms, definitions of those terms and relationships between those terms. Having captured knowledge, an ontology can be used to create a shared understanding of the domain. It is possible to be an ontology without necessarily having all the components of a rich ontology. It is not the question of whether an artifact is an ontology, but whether it is a rich or poor ontology.

What is possible with the ontology is determined not only by its content, but the structure of relationships in which that content or terms are held. Rich ontologies can offer much more than mere controlled vocabularies, by means of the structure of relationships in those terms or concepts are held. Such ontologies can improve querying annotations, creating annotations and using knowledge in annotations during analysis of biological entities. The richer an ontology, the more versatile it is likely to behave. The primary aim of an ontology is to create a shared understanding of a domain; first between humans and then an understanding that can allow machine processing. As the amount of data in biology increases, computational support for human analysis of that data becomes more and more important. Sufficiently rich ontologies, that are flexible and versatile, can have an important role in supporting this analysis by making the knowledge in resources such as SWISS-PROT computationally accessible.

### References

1. Bairoch A, Apweiler R. 1999. The SWISS-PROT Protein Sequence Data Bank and its supplement TrEMBL in 1999. *Nucl Acids Res* **27**: 49–55.
2. Bates M. 1998. Indexing and access for digital libraries and the internet: human database and domain factors. *J Am Soc Info Sci* **49**: 1185–1205.
3. Blackburn S. 1996. *The Oxford Dictionary of Philosophy*. Oxford University Press: Oxford.
4. Borgida A. 1995. Description logics in data management. *IEEE Trans Knowledge and Data Engineering* **7**: 671–782.
5. Etzold T, Ulyanov A, Argos P. 1996. SRS: Information retrieval system for molecular biology data banks. *Methods Enzymol* **266**: 114–128.
6. Jones DM, Visser PRS, Paton RC. 1998. Addressing biological complexity to enable knowledge sharing. In *AAAI'98 Workshop on Knowledge Sharing Across Biological and Medical Knowledge-based Systems*.
7. R, Goble CA, Bechhofer S. 2000. Ontology-based knowledge representation for bioinformatics. *Briefings Bioinformat* **1**: 398–416.
8. The Gene Ontology Consortium. 2000. Gene Ontology: Tool for the unification of biology. *Nat Genet* **25**: 25–29.
9. Uschold M, King M, Moralee S, Zorgios Y. 1998. The enterprise ontology. *Knowledge Eng Rev* **13**: 31–89.

Copyright © 2002 John Wiley & Sons, Ltd.

## References

[PDF_00382] Etzold T., Ulyanov A., Argos P. (1996). SRS: information retrieval system for molecular biology data banks.. Methods Enzymol.

[PDF_00389] Stevens R., Goble C. A., Bechhofer S. (2000). Ontology-based knowledge representation for bioinformatics.. Brief Bioinform.

